# Co-infection with a viral pathogen (Theiler’s murine encephalomyelitis virus) tended to improve host tolerance but significantly enhanced resistance to *Heligmosomoides bakeri*

**DOI:** 10.1007/s11259-026-11217-0

**Published:** 2026-05-01

**Authors:** Insani H. Zulfa, Rennos Fragkoudis, Andrea Doeschl-Wilson, Jos G. M. Houdijk, Margo Chase-Topping, Richard I. Bailey, Spiridoula Athanasiadou

**Affiliations:** 1 Scotland’s Rural College, West Mains Road, Edinburgh, Scotland, EH9 3JG UK; 2 University of Edinburgh, Edinburgh, Scotland, EH9 3BF UK; 3 Roslin Institute, University of Edinburgh, Midlothian, Scotland, EH25 9RG UK; 4 University of Lodz, Łódź, 90-237 Poland; 5 Faculty of Animal Science, Universitas Gadjah Mada, Yogyakarta, 55281 Indonesia

**Keywords:** Resistance, Tolerance, Helminths, Co-infection, *Heligmosomoides bakeri*, Theiler’s murine encephalomyelitis virus

## Abstract

The host’s ability to clear parasites (resistance) and their capacity to reduce the impact of a given parasite burden (tolerance) is often described under conditions of a single pathogen challenge. We developed a mouse infection model to investigate the impact of co-infection on host resistance and tolerance to *Heligmosomoides bakeri*. C57BL/6 mice were infected with the helminth parasite *H. bakeri* and Theiler’s murine encephalomyelitis virus (TMEV), that both reside in the small intestine. Two infection protocols were used to also investigate the impact of the order of pathogen administration on host resistance and tolerance (H-V protocol: helminth first and V-H protocol: virus first). Non-infected controls, *H.bakeri* only and TMEV only infected animals were also included. Our data showed that co-infection in the H-V protocol, resulted in significantly improved resistance to *H.bakeri*, as measured by 30% lower number of *H. bakeri* recovered (*p* = 0.023) and a 57% reduction in eggs in colon (*p* = 0.035) compared to mice infected with *H. bakeri only*. It also led to a 39% improvement in tolerance against *H. bakeri* infection compared to mice infected with *H. bakeri only* (*p* = 0.052). The positive impact on the resistance to *H. bakeri* was evident but less pronounced in mice in the V-H protocol. Our hypothesis that resistance and tolerance of animals are penalised from increased pathogen load is rejected with important implications for disease control.

**Supplementary Information:**

The online version contains supplementary material available at 10.1007/s11259-026-11217-0.

## Introduction

There are two possible mechanisms that hosts use to defend themselves against pathogen infection, resistance (their ability to clear the pathogen) and tolerance (their ability to suppress the impact of the pathogen on productivity and fitness) (Medzhitov 2007; Svensson and Råberg 2010; Hayward et al. 2014; Lough et al. 2015). The measurements of resistance and tolerance are fundamentally different. Resistance is measured as a function of within-host pathogen load (per individual or per type of tissue); in general, individuals with low pathogen load (under conditions of similar exposure to/administration of the pathogen) are considered more resistant compared to individuals with high load (Råberg et al. 2009). On the other hand, tolerance is commonly expressed as the slope of a regression of host fitness or performance traits, against within-host pathogen load; under similar exposure to/administration of the pathogens, a steeper negative slope is indicative of a more tolerant individual (Simms 2000; McNew et al. 2019). Tolerance is not often measured in infection studies; whilst some studies report the impact of infection on performance, very few studies explicitly consider the relationship between performance and within-host pathogen load. Quantifying host tolerance to infection, is equally beneficial as determining their resistance, as tolerant individuals will maintain high performance, even under conditions of heavy infection.

In parasitology, many studies on host resistance and tolerance to date have been limited to single pathogen infections, whereas in reality hosts are often infected with more than one pathogen at any one time (Cox 2001; Susi et al. 2015). To this date, the impact of co-infection on host resistance and tolerance to parasites, and the factors that may be affecting those are poorly investigated and understood. It has been suggested that the types of pathogens involved, the timing of infection, the pathogen niche, and possibly their mode of replication within the host (Furze et al. 2006) may be affecting the impact, but the pattern is not consistent. A better understanding of the impact of co-infection on host defence mechanisms is vital for effective disease control.

In the present study we aimed to quantify the impact of co-infection and the order of pathogen infection, on the resistance and tolerance of mice parasitised with the helminth *Heligmosomoides bakeri*, as a model for livestock that are often under multiple pathogen challenge. *H. bakeri* is naturally found in mice and has been used as a model of chronic intestinal nematode infection in humans and livestock (Behnke et al. 2009). Due to their slow response to *H. bakeri* infection, C57BL/6 mice are a good model for parasitic nematode infection in livestock (Athanasiadou et al. 2015). The second pathogen is Theiler’s murine encephalomyelitis virus (TMEV). TMEV is an RNA virus with a single strand positive sense genome belonging to the Cardiovirus genus of the *Picornaviridae* family. TMEV is a neurotropic, enteric rodent pathogen transmitted through the oral-faecal route. Although cell tropism in the gastrointestinal tract is unknown for TMEV, there is evidence to suggest TMEV is closely associated with the mucus at the surface of the intestine and has been shown to be bound to goblet cells (Tsunoda et al. 2009). Both *H. bakeri* and TMEV can result in subclinical disease (Oleszak et al. 2004; Maizels et al. 2012), which is one of the main reasons for reduced productivity and efficiency in livestock (Houdijk et al. 2017). Previous evidence has shown that intestinal helminth -virus co-infection is detrimental to host resistance, as it has been shown to enhance infection and transmission of enteric viruses, such as murine norovirus and murine astrovirus (reviewed by Desai et al. 2021); not much evidence is available on the impact of the viral pathogen on the helminth load. Using TMEV as a model viral pathogen, allowed us to test our hypothesis that co-infection with an intracellular pathogen at the same niche as *H.bakeri*, will detrimentally affect host responses, resistance and tolerance to *H.bakeri.* We also hypothesised that the order of pathogen administration will have an impact on the manifestation of host’s resistance, as this is measured by parasitological parameters.

## Materials and methods

### Experimental animals and housing

The animal experiment was approved by Scotland’s Rural College (SRUC), UK, Ethical Review Committee (ED AE 09/2017) and carried out under Home Office authorization (PPL 60/4935). A total of 65 female C57BL/6 mice (approximately 5 weeks old as determined by the breeding company) were provided by Charles River housed in a room with a temperature of 21 ± 1 °C and 12:12 h light: dark cycle (07:00–19:00 h). Female mice were selected for this study as they tend to be less susceptible to helminth infections compared to males (Sellau et al. 2024) and thus the outcomes of the study were likely to have wider implications. Mice were group housed in solid bottomed cages (5 mice per cage) with fresh bedding material provided weekly, with shredded paper as environmental enrichment. Mice were offered *ad libitum* a standard maintenance diet (14% crude protein; Special Diet Services, Lillico Biotechnologies, UK).

### Infection protocol and experimental design

The experiment consisted of two biological replicates (i.e. separate cages) for each of the treatment groups described below.

**Fig. 1:**
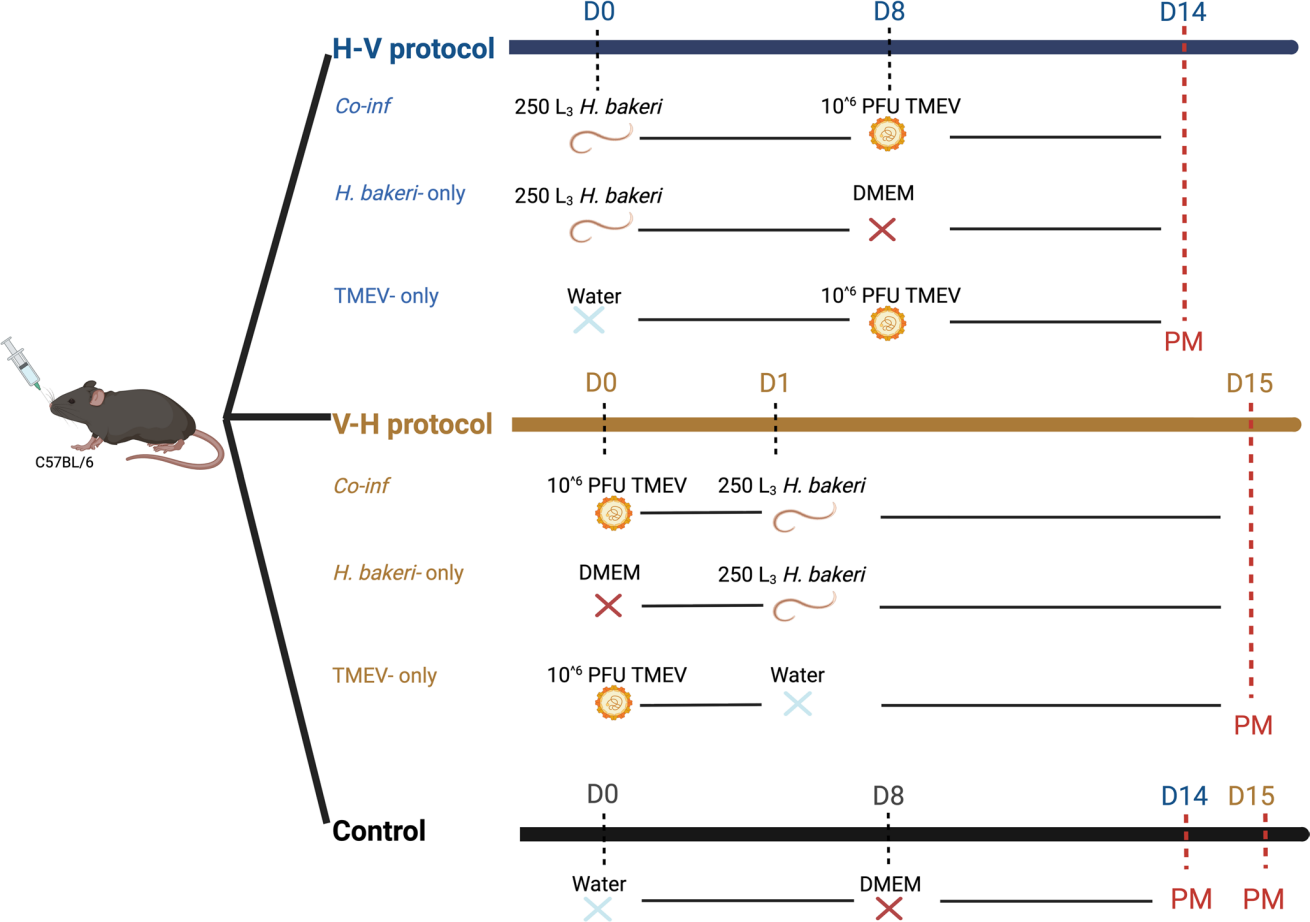
Experimental design. C57BL/6 mice were subjected to two co-infection protocols. In the H-V protocol (*n* = 10) mice were administered orally 0.2 ml of 250 *H. bakeri* 3rd stage infective larvae (L3) or water (control) at day 0 (D0) followed by 0.2 ml of 10^^6^ PFU TMEV or DMEM (Dulbecco s Modified Eagles Medium) at day 8 (D8). In the V-H protocol (*n* = 10), mice were administered orally 0.2 ml of 10^6 PFU TMEV or DMEM at day 0 (D0) followed by 0.2 ml of 250 *H. bakeri* 3rd stage infective larvae (L3) or water at day 1 (D1). In the control group, mice (*n* = 5) were administered orally 0.2 ml of water at D0 and DMEM at D8. Mice were euthanised as per protocol on days 14 or 15 post initial pathogen challenge

Two infection protocols were implemented to determine whether the order to pathogen exposure had any effect on the impact of co-infection on resistance and tolerance of mice (Fig. 1). In H-V infection protocol, *H. bakeri* (H) was administered first followed by the TMEV (V) challenge, whereas in V-H infection protocol, TMEV was administered first followed by the *H. bakeri* challenge. Within each infection protocol, mice were exposed to one of four infection challenges: each pathogen alone (*H. bakeri-only*; or TMEV-only), both pathogens (co-infection, *Co-inf*) or sham infected controls (sham Con). *H. bakeri* infection was administered as a single dose of 250 L_3_ suspended in 0.2 mL water via oral gavage (Houdijk and Bünger 2007). TMEV infection was administered as a single dose of an avirulent TMEV BeAn strain (curtesy of Dr Fragkoudis) using 10^^6^ plaque forming units (p.f.u) suspended in 0.2 ml Dulbecco’s Modified Eagles Medium (DMEM) by oral gavage (Kang et al. 2007). The dose of TMEV and *H. bakeri* were chosen to achieve a subclinical level of infection, which has been reported to affect mouse growth (Oleszak et al. 2004; Kang et al. 2007; Houdijk and Bünger 2007).

The detailed infection protocols are seen in Fig. 1. At day 0, mice in the H-V infection protocol received either *H. bakeri* (*Co-inf* and *H. bakeri-*only treatment) or a sham infection (TMEV-only and sham Con); at day 8 mice in co-infection and TMEV treatments were challenged with TMEV. *H. bakeri-*only mice were given Dulbecco s Modified Eagles Medium (DMEM) at day 8. At day 0, mice in the V-H infection protocol received either TMEV (*Co-inf* and TMEV-only treatment) or DMEM (*H. bakeri*-only treatment); at day 1 in this protocol, mice in *Co-inf* and *H. bakeri*-only treatment were challenged with *H. bakeri*.

The rationale for the timing of the administration of the second pathogen in the co-infection groups was associated with the establishment of the first infection. In H-V infection protocol, we administered TMEV at day 8 post *H. bakeri* infection in the *Co-inf* mice, since *H. bakeri* is expected to re-emerge in the small intestine 8 days post-infection (Allen and Maizels 2011). In V-H infection protocol, we administered *H. bakeri* at day 1 post TMEV infection in the *Co-inf* mice, since the peak of TMEV viraemia has been reported to be 1 to 3 days post infection (Oleszak et al. 2004). Thus, within both infection protocols, both pathogens were expected to be present in the small intestine simultaneously. Twenty-four hours post the oral TMEV challenge in both protocols (9 d.p.i in H-V and 1 d.p.i in V-H infection protocol) two mice (per replicate) were sacrificed to quantify virus (or viral RNA) presence in the blood/tissue. The remaining mice were euthanised for parasite recovery 14 days post *H. bakeri* infection, in each protocol. This meant that in H-V, mice were euthanised at day 14 post infection, whereas in V-H protocol mice were sacrificed at day 15. At day 14 post *H. bakeri* infection, adult worms were expected to be in the small intestine, as they typically emerge 8 days post infection (Maizels et al. 2012); in the C57BL/6 mice day 14 post infection has been observed to coincide with peak infection (Reynolds et al. 2012).

### Measurements and sample collection

#### Body weight measurement

Individual body weight (BW) data and feed refusals per cage were collected every 2 or 3 days throughout the experiment. On each of these days, feed refusals were weighed out and fresh feed added was weighed in per cage.

#### Eggs in colon (EIC) and worm burdens (*H. bakeri*)

During PM dates as these are shown in the experimental protocol, mice were humanely killed via CO2 inhalation and dissected for sample recovery. The small intestine was cut open and placed in a tube with PBS, followed with 37 °C incubation for 3 h to allow worms to migrate out of the tissue. The tissue and recovered worms were then stored in a 5% formalin solution until quantification. Male and female worms were quantified; the colon content was collected and weighed and a faecal egg count was performed on the colon content using a modified flotation technique (Christie and Jackson 1982). The colon egg count was then multiplied by the weight of the colon content to account for dilution effects arising from individual variation, such as variation in feed intake or egg excretion patterns between animals. The data were expressed as number of eggs in colon (EIC). The EIC, male, female, per capita fecundity and total worm counts were used as indicators of host resistance against *H. bakeri*.

#### Plaque assays and PCR (TMEV)

To confirm that the viral infection was successful, blood and tissue samples were collected at post-mortem (PM). It was important to demonstrate that oral inoculation of the TMEV leads to infection and persistence of the virus in the small intestine, whilst adult *H.bakeri* were also present there. Plaque assays were performed to quantify TMEV virus load in the blood (viraemia) (Kang et al. 2007); all samples were analysed in duplicate (technical replicates) and the assay was repeated twice. In addition, brain and small intestine samples collected at PM were stored in RNAlater for RNA extraction, to investigate the presence of viral RNA in the tissue, in the co-infection and TMEV-only groups. RNA extraction was performed using Qiagen RNeasy mini kit (CAT no 74104, Qiagen, UK) following the manufacturer’s guideline. RNA quantities and A260/280, A260/230 ratios were measured to assess RNA purity using Nanodrop spectrophotometer (Nanodrop technologies Inc., USA). cDNA synthesis was performed using Thermo Scientific Verso cDNA synthesis kit (CAT no AB1453A, Thermo Scientific, UK) following manufacturer’s guideline with oligodt used as RT-primers. Thermal cycle profile for cDNA synthesis was 42˚C for 30 min following 95˚ for 2 min. PCR was carried out using Thermo Scientific Platinum™ II Hot-Start PCR Master Mix (Cat no 14000013, Thermo Scientific, UK). Primers have been previously published (Uhde et al. 2016) with forward primer GACTAATCAGAGGAACGTCAGC and reverse primer GTGAAGAGCGGCAAGTGAGA (amplicon size 129 bp). Thermal cycle profile for standard PCR was 95˚C for 3 min, 40 cycles of 98˚C for 15 s, 60˚C for 20 s, and 72˚C for 1 min, and elongation at 72˚C for 20 s. PCR products were visualised by gel electrophoresis.

#### Measure of tolerance to *H. bakeri*

As mentioned earlier, tolerance is measured as the slope of a linear regression of host performance against the within-host, pathogen burden; a steeper negative slope indicates lower tolerance (faster decrease in performance per unit change of pathogen burden) (Lough et al. 2015). The performance traits used to estimate tolerance in this study were carcass weight at PM and body weight (BW) gain, estimated as the difference between final BW measured at D14/D15, and initial BW. Previous studies have shown that although BW is the most studied performance trait, it is dependent on gut fill fluctuations, unlike carcass weight (Athanasiadou et al. 2015). Total worm counts and EIC were used as measures of parasite burden in the regression analyses for tolerance. More specifically, total worm counts, as a direct measure of parasite load, was used as the direct independent variable for tolerance estimates, whereas EIC was used as indirect independent variable. Hence, four different tolerance estimates were derived from this study based on regression of carcass weight against total worm burden, carcass weight against EIC, BW gain against total worm burden, and BW gain against EIC.

#### Statistical analysis

Animal performance (measured as BW gain) was analysed in two different ways: within each infection protocol and across protocols. To assess the effect of co-infection over time, the experiment was divided into three periods: pre-infection, mid-infection and post-infection. The pre-infection period was determined as the period prior to any infection treatment (prior to day 0). Mid-infection was determined as the period following the first infection treatment (day 0 to day 8 within H-V protocol and day 0 to day 1 within V-H protocol), whereas as post-infection was the period following the second infection treatment (day 9 to day 14 within H-V protocol and day 2 to day 15 within V-H protocol). To determine the impact of co-infection on performance, BW gain was analysed using a general linear mixed model. Fixed effects included in the model were the protocol (2 protocols, i.e. H-V and V-H), mode of infection (4 modes, i.e. *Co-inf*, *H. bakeri*-only, TMEV-only and sham Con), and period (3 periods; pre-infection, mid-infection and post-infection), as well as all possible two- and three-way interactions. In addition, pre-infection BW was fitted as covariate in the model for BW gain, and mice identity nested within cage was fitted as a random effect as a conservative approach, to account for potential individual-level randomness, thus preventing Type 1 errors.

To determine the impact of co-infection on resistance of mice to *H. bakeri*, a linear model implemented in the R-package *LME4* was fitted for EIC, worm counts and per capita fecundity in parasitised mice as the response variables. In H-V protocol, worm count data from mice sacrificed on day 9 d.p.i (for TMEV detection) were included in the analysis; there were no parasitological outcomes from mice sacrificed 1 d.p.i. in the V-H protocol. EIC, worm counts, and per capita fecundity were log-transformed prior to analysis to account for skewness in their distribution and normalize model residuals. Fixed effects included in the models were: protocol (H-V and V-H); mode of infection (two modes; *Co-inf* and *H. bakeri*-only), and their interaction. AIC was used to determine the most suitable model for the analysis; the model with the lowest AIC value was selected for each variable. For EIC, *lm* model with the fixed effects (protocol and mode of infection) and interaction (protocol*mode of infection) without random effect was deemed fit as final model. The final model further was checked for goodness-of-fit using ‘check_model’ function from the performance package (Lüdecke et al. 2021) (Supplementary file S2-model fit). For worm counts and per capita fecundity, *lm* model with the fixed effects (protocol and mode of infection) without interaction and random effects were deemed fit as final model. When there were significant main effects (e.g.in EIC), *posthoc* analysis was conducted using the ‘emmeans’ function from the emmeans package (Lenth 2024), adjusting for multiple comparisons. For transformed data, results are presented as back transformed means with 95% confidence intervals (CI).

Regression models implemented in the R-package *lmer* were used to assess the impact of co-infection on tolerance of parasitised mice to *H. bakeri* infection. The regression model used either carcass weight or BW gain as dependent variable, mode of infection (two modes; *Co-inf* and *H. bakeri*-only) as fixed effect, and parasite burden (log10 (WC + 1) and log10 (EIC + 1) as well as initial BW, as covariates. Specific tolerance slope estimates were obtained by including the interaction term mode of infection × parasite burden as additional fixed effects in the final models, where non-significant variables had been removed from the final model. AIC was used to determine the model selection, whereas, the final model was checked for goodness-of-fit using the performance package (Lüdecke et al. 2021). As infection protocol did not have an impact on tolerance, data from the two co-infection protocols (H-V and V-H) were pooled for the tolerance analyses Significant differences between the tolerance slope estimates based on the F-test statistics provided evidence on the impact of co-infection vs. single parasite infection on tolerance against *H. bakeri*.

## Results

### No viraemia, but viral RNA was detected in the small intestine and the brain of TMEV infected mice

No viral plaques were detected in the assays performed with blood samples recovered from both protocols at time points that coincided with day 1 post viral challenge (days 1 and 9 in protocols V-H and H-V respectively), indicating that hosts’ viraemia was below the limit of detection of the assay.

**Fig. 2:**
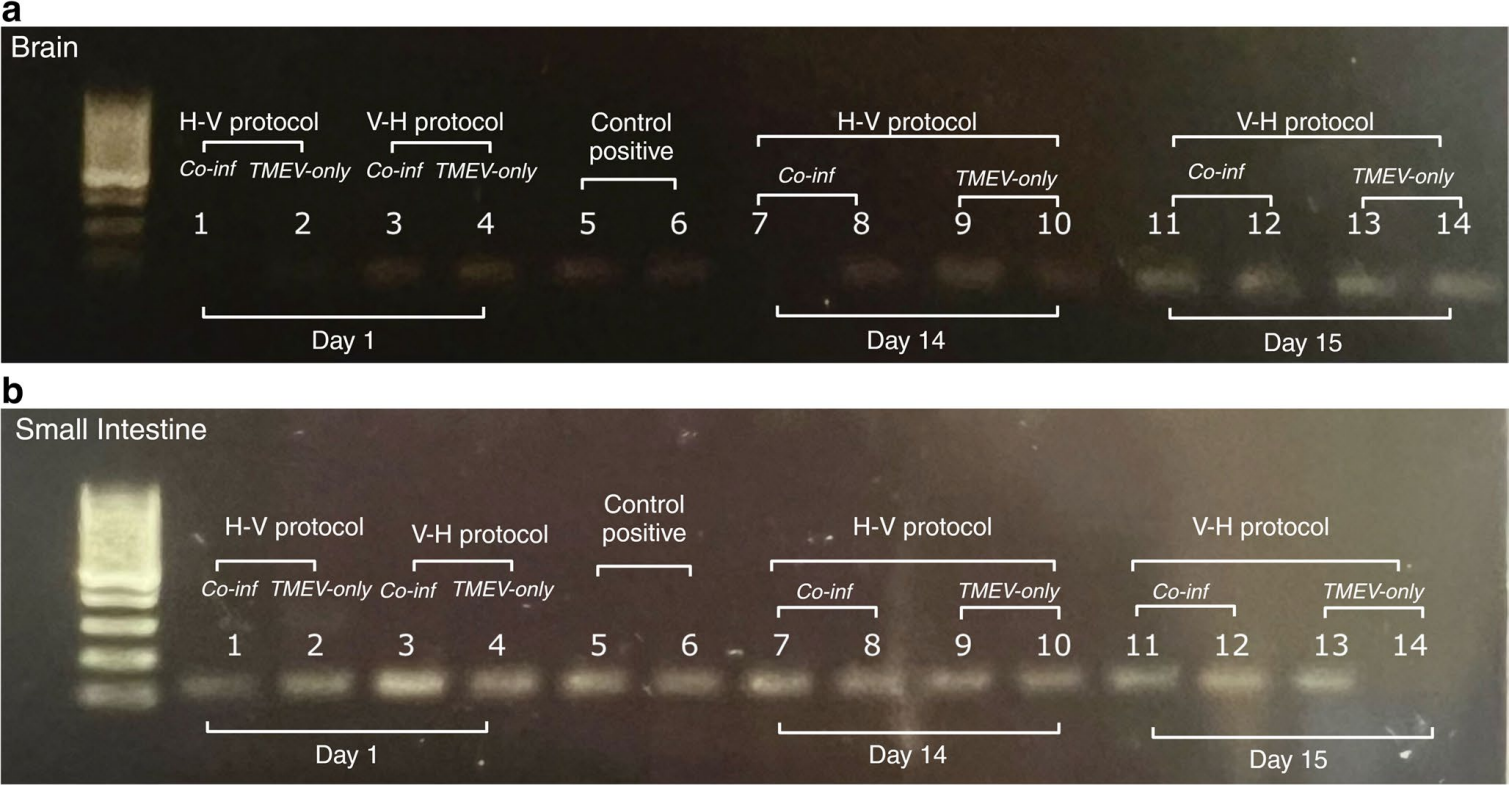
Gel electrophoresis of PCR products from the brain (**A**) and the small intestine (**B**) samples of mice infected with TMEV-alone or co-infected with *H.bakeri*. Day 1 samples were pooled following post-mortem (two mice per cage was euthanized, four mice in total per infection protocol). Day 14/15 samples were pooled following final post-mortem (remaining mice were euthanized). Positive controls derived from TMEV infected cell cultures. The presence of a band indicates the presence of viral RNA in the specific pooled sample

Most of previously published studies perform intracerebral or intraperitoneal inoculation of TMEV; we demonstrated that oral inoculation of TMEV resulted in the virus being detected in the small intestine (within the first 24 h) and/or brain (days 14/15) of inoculated mice. Figure 2B shows that very little viral RNA was detected in the brain within the first 24 h post inoculation.

Within the H-V protocol (Fig. 2), at day 1 post virus infection (9 DPI), viral RNA was detected in the small intestinal samples from both TMEV-only and *Co-inf* treatment; viral RNA was not detected in the brain of animals at day 1 post virus infection (Fig. 2B; Figs. 1 and 2). At day 14 DPI, TMEV RNA was detected in small intestine and brain samples from TMEV-only and *Co-inf* animals.

Within the V-H protocol (Fig. 2), at day 1 post virus infection (1 DPI), TMEV RNA was detected in the small intestine and brain samples from both TMEV-only and *Co-inf* animals. At day 15 DPI, TMEV RNA was detected in small intestine and brain samples from TMEV-only and *Co-inf* animals. The lack of viral RNA in a single intestinal sample (sample 14; Fig. 2A), maybe due to technical error or viral RNA clearance from the small intestine, as it was detected in the brain of the same animals at the end of the study (sample 14; Fig. 2B).

### Co-infection did not affect BW gain of mice

There was no significant difference in BW gain of mice throughout the whole experimental period in any of the two protocols (H-V or V-H infection protocols). Similarly, mice that received the co-infection challenge, had similar BW gain across the two protocols (0.142 g for H-V protocol and 0.141 g for V-H protocol; S.E.D. 0.548, *p* > 0.1),

### Co-infection resulted in mice having lower parasite load than those in H. bakeri-only infection

**Fig. 3:**
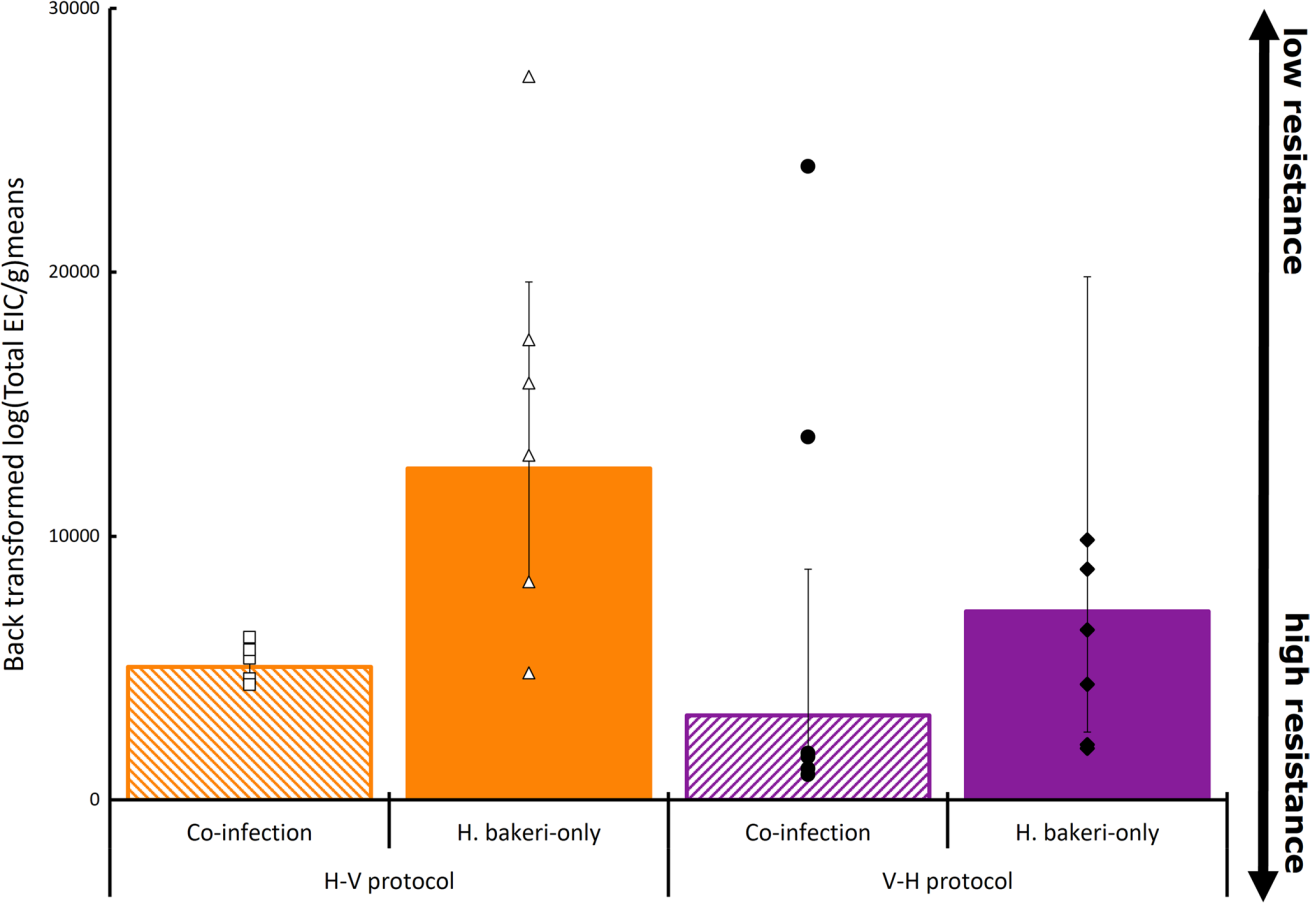
Back transformed least square means of eggs in colon (EIC; eggs/g faeces) of mice infected with 250 *H. bakeri* infective larvae alone or in TMEV co-infection, in two infection protocols (H-V and V-H; *n* = 6). Individual mouse EIC are presented in markers, and the bars represent lower and upper confidence intervals

**Fig. 4:**
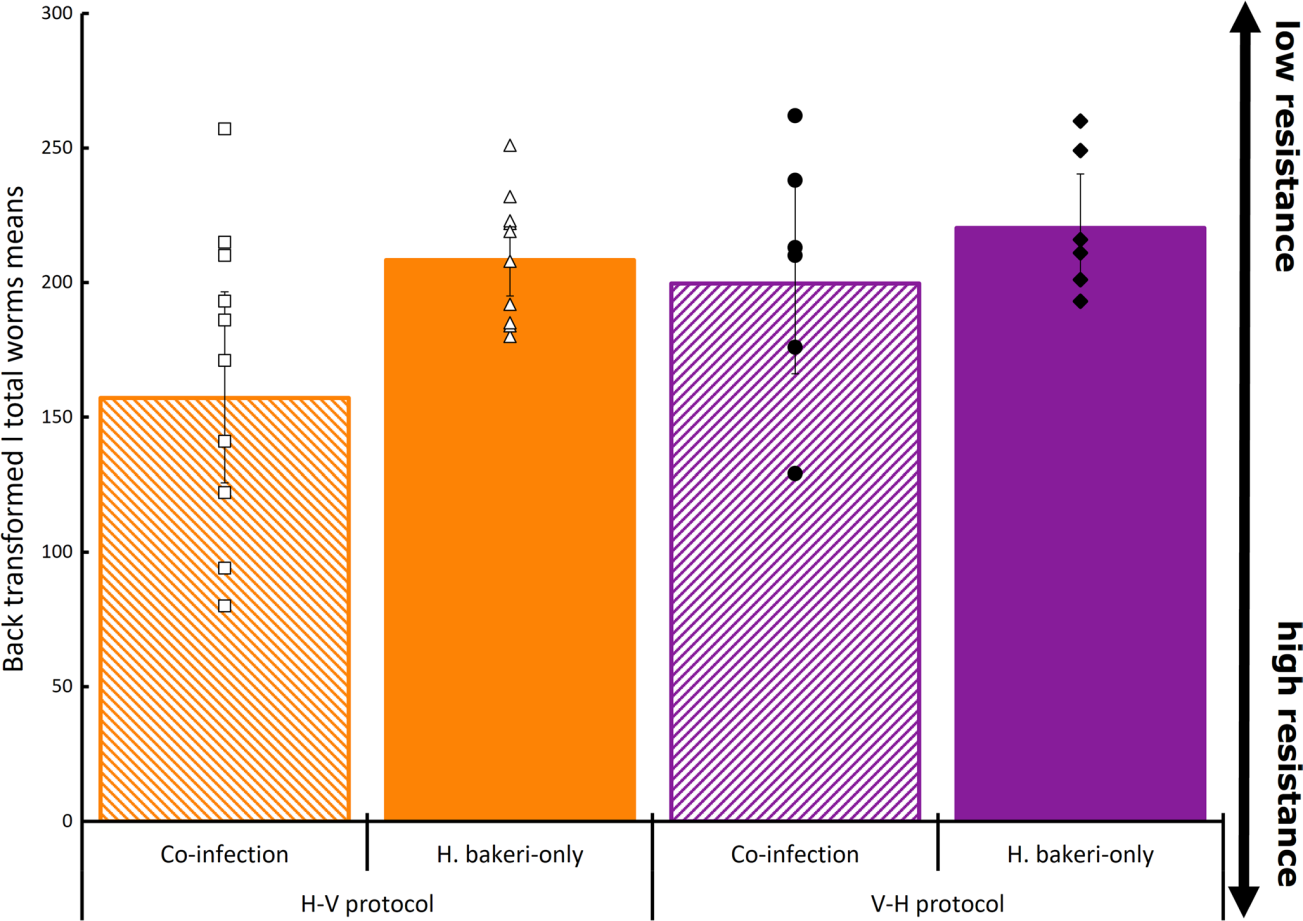
Back transformed least square means of female (**A**), male (**B**) and total worm (**C**) numbers of mice infected with 250 *H. bakeri* infective larvae alone or in TMEV co-infection, in two infection protocols (H-V, *n* = 10; V-H, *n* = 6). Individual mouse female, male and total worm numbers is presented in markers, and the bars represent lower and upper confidence intervals. Total worm burdens also contain worms that could not be identified as male or female (e.g. partial worms)

**Fig. 5:**
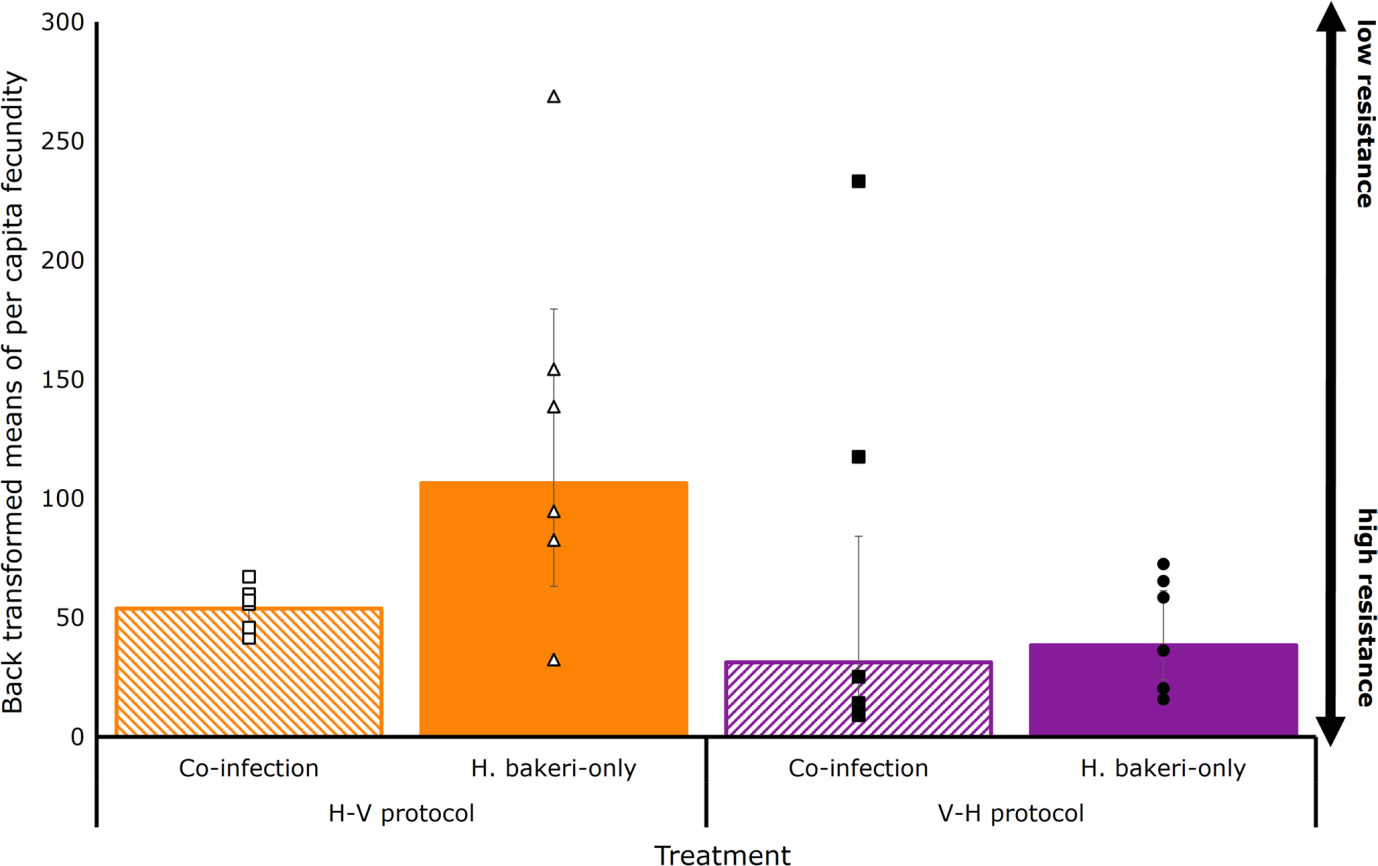
Back transformed least square means of per capita fecundity of mice infected with 250 *H. bakeri* infective larvae alone or in TMEV co-infection, in two infection protocols (H-V and V-H; *n* = 6). Individual mouse per capita fecundity is presented in markers, and the bars represent lower and upper confidence intervals

Statistical results from the final model of host resistance traits are presented in Table 1. There was a significant interaction between protocol and mode of infection for EIC (*p* = 0.037). Within the H-V infection protocol, co-infected mice had 57% less eggs in their colon compared to *H. bakeri*-only mice (backtransformed means with 95% lower and upper CI: 5,051 (509–567) vs. 12,563 (4,518-7,057) eggs respectively, *p* = 0.035; Fig. 3). Within the same protocol, co-infected mice had fewer male (48 (11–15) for *Co-inf* and 65 (5–6) for *H. bakeri*-only), female (93 (16–20) for *Co-inf* and 122 (9–10) for *H. bakeri*-only; and total (157 for *Co-inf* and 208 for *H. bakeri*-only; *p* = 0.023; Fig. 4) worms than mice which received *H. bakeri*-only. Fecundity was 45% lower in co-infection treatment compared to *H.bakeri*-only (53 (6–7) eggs/female worms for *Co-inf* and 106 (43–73) eggs/female worms for *H. bakeri*-only; *p* = 0.015; Fig. 5).

**Table 1:** Outcomes of the linear model analysis (R-package *LME4)* testing the effects of protocol and mode of infection, and their possible interactions on female, male and total worms, EIC and per capita fecundity

Variable		Estimate	SE	F value	*p*-value	(95%CI)	Residual SE	dF
Total worms	Protocol	0.174	0.091	1.918	0.065	−0.01 −0.36	0.248	29
	Mode of infection (Treatment)	0.402	0.087	4.573	< 0.001	0.22–0.58		
Female worms	Protocol	0.117	0.099	1.183	0.247	−0.09 −0.32	0.273	29
	Mode of infection (Treatment)	0.426	0.096	4.422	< 0.001	0.23–0.62		
Male worms	Protocol	0.139	0.085	1.640	0.111	−0.03 −0.31	0.234	29
	Mode of infection (Treatment)	0.368	0.0825	4.468	< 0.001	0.20–0.54		
EIC	Protocol	−0.337	0.125	−2.708	0.0135	−0.60 - −0.08	0.216	29
	Mode of infection (Treatment)	1.263	0.125	10.151	< 0.001	1.00–1.52		
	Protocol ⋅ mode of infection	−0.392	0.176	−2.229	0.037	−0.76 - −0.03

Within the V-H infection protocol, a similar but not statistically significant trend was observed; co-infected mice had on average 3,209 (2,031 − 5,534) eggs in their colon whereas *H. bakeri*-only had 7,147 (1,860-3,105) eggs in their colon (*p* = 0.118; Fig. 3). Similarly, the number of total (199 (34–40) for *Co-inf* and 220 (18–20) for *H. bakeri*-only; *p* = 0.105), male (69 (10–12) for *Co-inf* and 71 (3–4) for *H. bakeri*-only; *p* = 0.158), and female (104 (21–26) for *Co-inf* and 122 (16–18) for *H. bakeri*-only; *p* = 0.098; Fig. 4) worms, along with the fecundity (31 (19–52) vs. 38 (14–22) eggs/female worms for *Co-inf* vs. *H. bakeri*-only respectively; *p* = 0.216) did not statistically differ from mice on *H. bakeri* only infection (Fig. 5). There was no difference in worm numbers for the co-infection groups between the two infection protocols (157 for H-V and 199 for V-H; S.E.D. 11.27; *p* = 0.262).

### *H. bakeri*-only infected mice tended to be less tolerant compared to mice under co-infection

**Fig. 6:**
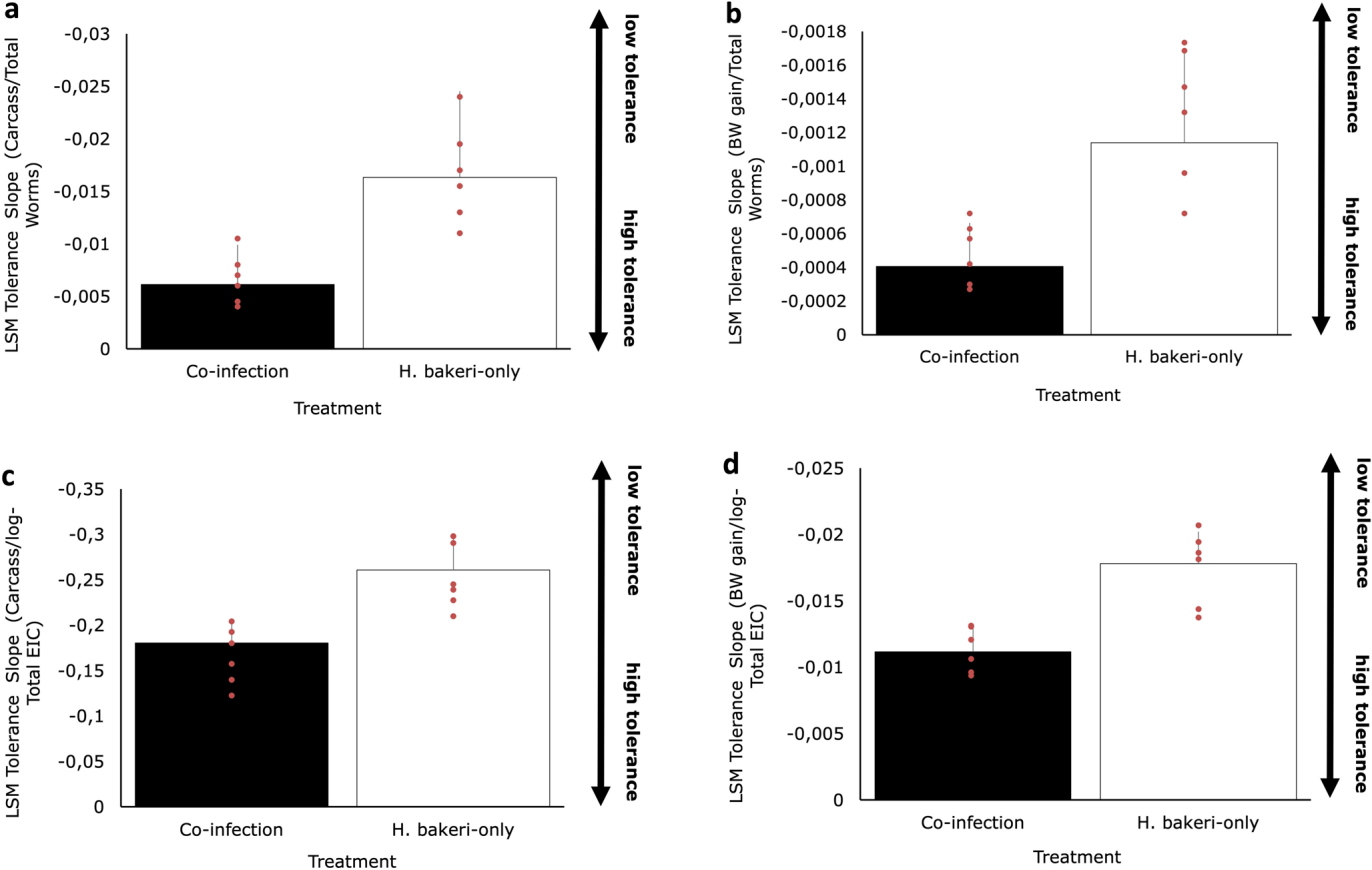
Estimates of tolerance of mice infected with 250 *H. bakeri* infective larvae alone or in TMEV co-infection, in two infection protocols (H-V and V-H) against *H. bakeri*, as estimated via regression analyses of performance vs. within-host parasite burden. (**A**) Carcass weight vs. Total worms; (**B**) BW gain vs. Total worms; (**C**) Carcass weight vs. EIC; (**D**) BW gain vs. EIC. Markers indicate individual animals

The tolerance analysis generally produced negative least square mean (LSM) tolerance slope estimates that were significantly below zero (*P* < 0.05), regardless of the mode of infection and the performance and parasite burden traits used in the analyses (Fig. 6). This indicates that mice are not fully tolerant to *H. bakeri* infection. Furthermore, *H. bakeri-*only infected mice were consistently found to be less tolerance (i.e. had steeper tolerance slope estimates) than co-infected mice (Fig. 6). The differences were most pronounced when total worm counts were used as measures of parasite burden, bordering statistical significance. LSM tolerance slope estimates using carcass weight as response variable were − 0.0062 ± 0.0037 for *Co-inf* and − 0.0163 ± 0.0082 for *H. bakeri*-only (*p* = 0.052; Fig. 6A), which equates to 39% improvement in co-infected mice; those estimates for BW gain as response variable were − 0.0004 ± 0.0002 for *Co-inf* and − 0.0011 ± 0.0005 for *H. bakeri*-only; (*p* = 0.058; Fig. 6B).

Similar, though less pronounced differences in tolerance were observed based on carcass weight against EIC (*Co-inf* − 0.181 ± 0.027 vs. *H. bakeri* only − 0.261 ± 0.035; *p* = 0.071; Fig. 6C) and BW gain against EIC (*Co-inf* − 0.011 ± 0.002 vs. *H. bakeri* only − 0.018 ± 0.009; *p* = 0.098 Fig. 6D).

## Discussion

To the best of our knowledge this is the first study where the resistance and tolerance of a host known to be relatively susceptible to an intestinal helminth was quantified in the presence of a viral pathogen. Although a plethora of studies have previously described co-infection models (Desai et al. 2021), very few have investigated the dynamics between intestinal helminths and viruses (e.g. Osborne et al. 2014) and those have mostly quantified the impact of the helminths on the viral load, rather than the impact of the viral challenge on the helminths and host defences. Our hypothesis was that during co-infection with a viral pathogen that resides at the same niche, mice exposed to *H. bakeri* will experience a bigger penalty on their resistance and tolerance compared to mice that are exposed to a single *H. bakeri* infection. In contrast, we observed that administration of TMEV following an established *H. bakeri* infection resulted in a significant positive impact on host resistance to *H.bakeri* as measured by EIC (*p* = 0.035), worm burden (*p* = 0.023) and per capita fecundity (*p* = 0.015). We also observed that co-infected mice tended to have better tolerance response against *H. bakeri* infection, compared to *H. bakeri-*only mice (*p* = 0.052). Our initial hypothesis is therefore rejected. Furthermore, we found that the order of pathogen administration has an impact on the manifestations of host defence strategies as the administration of TMEV following an established *H. bakeri* infection resulted in a greater positive impact on resistance compared to administration of TMEV before the *H.bakeri* infection.

In line with previous studies of parasite resistance (Raberg et al. 2007; Hayward et al. 2014; Athanasiadou et al. 2015), which is defined as the ability of the host to clear the pathogen, we used EIC and worm burden as indirect and direct indicators of resistance respectively. Within the H-V protocol of infection, co-infected mice excreted significantly fewer eggs and had significantly lower worm burden compared to mice that received *H. bakeri*-only. The outcome followed a similar, although not statistically significant pattern in the V-H protocol of infection. C57BL/6 mice are in general categorized as slow responders against *H. bakeri* infection, with a slowly induced Th2-immune response, which results in parasite expulsion within 8–20 weeks post challenge (Reynolds et al. 2012). The improved resistance to *H. bakeri* observed could have been attributed to reduced *H. bakeri* establishment in the small intestine or early worm expulsion. Previous studies have reported mixed impact of co-infection on resistance to pathogens. Administration of Influenza A virus during a patent *Litomosoides sigmodontis* infection resulted in increased host susceptibility to the virus, higher lung viral titre and clinical signs earlier, compared to single infected mice; however, there was no impact on adult worm counts (Hardisty et al. 2022). On the other hand, host resistance to influenza A virus was enhanced in mice co-infected with *T. spiralis* as demonstrated by lower viral replication, compared to mice infected with a single pathogen (Furze et al. 2006). However, no impact was measured on the helminths. To the best of our knowledge, previous studies investigating co-infection at the same niche, such as Osborne et al. 2014;, have shown penalties of host resistance to the viral pathogen, whereas the impact on the helminths was never really measured.

The reasons underpinning the impact of co-infection on resistance remain contentious; previous studies have indicated that the observed phenotype may be associated with changes in immunity (Du Plessis et al. 2013; Karadjian et al. 2014). A reduction in *M. tuberculosis* load observed during co-infection with *Nippostrongylus brasiliensis* was associated with an early and increased activation of pulmonary CD4 T cells, T helper 1 (Th1) and Th2 cytokine production, and increased number of neutrophils and alveolar macrophages in the lung of mice *(*Du Plessis et al. 2013). A *L. sigmodontis* and *Plasmodium* spp co-infection resulted in reduced filarial load in mice, which concurred with increased circulation of pro-inflammatory cytokines (Karadjian et al. 2014). Although it is not always possible to establish causality, changes in immune response may be, at least partly, responsible for the shift in resistance observed. This suggestion is supported by the impact in the order of co-infection observed in our study, with mice in H-V protocol, experiencing a greater beneficial impact on their resistance. C57BL/6 mice are in general characterised as strong responders against TMEV (Oleszak et al. 2004) and they upregulate a strong type 1 immune response to protect against the virus. In the H-V protocol, the virus was administered during the period when larvae of *H. bakeri* re-emerge in the small intestine (day 8), which may have had an immunomodulatory effect on the host that resulted in improved resistance to *H.bakeri*. Indeed, in a recent study, TMEV infected macrophages were shown to upregulate mRNA expression of IL33 (Esmael and Petro 2023), a cytokine that has been associated with having a positive impact on the expression of immunity against helminth parasites (Hung et al. 2020). In addition, intracerebral inoculation with TMEV has been shown to increase intestinal permeability and numbers of CD4 + T cells in the lamina propria in the intestine of infected mice (Carrillo-Salinas et al. 2017); if oral administration of TMEV has a similar effect in the intestine, this may be responsible for the reduction in *H.bakeri* worm numbers observed in our study. Consequently, TMEV may have a role in modulating the immune response to *H.bakeri (*either via IL33 regulation or CD4 + T cells involvement*)*, as worms were already in the small intestine when TMEV was administered in the H-V protocol; this remains to be investigated.

Resistance to *H.bakeri* has been shown to be affected by changes in gut microbiota (Reynolds et al. 2012). Previous evidence has indicated that intracerebral TMEV administration induces changes in the relative abundance of gut microbiota in SJL/J mice 14 days post-inoculation (Carrillo-Salinas et al. 2017). If oral inoculation of TMEV results in changes in the microbiome, they may also have impacted host resistance to *H.bakeri*. In addition, pathogens residing at the same niche, which is the case in our study, may also be affected by space or resource competition. Resource competition has been illustrated in a malaria co-infection study (Huijben et al. 2011) where two different strains of malaria parasites, both targeting red blood cells of similar age and type, were in direct resource competition, resulting in lower parasitaemia for one of the two strains, thus limiting infection pressure in co-infected animals. Space competition has been hypothesised in an epidemiological study where parasitised humans showed significantly lower prevalence of natural giardia-helminth co-infections compared to single species infections, at areas where both pathogens were endemic (Blackwell et al. 2013). The presence of TMEV in the small intestine in our study at day 14 (H-V protocol) and day 15 (V-H protocol), as indicated via PCR, suggested that oral administration of TMEV resulted in the virus residing in the small intestine, as it has also been seen in wild mice (Clatch et al. 1985). The possibility that *H. bakeri* and TMEV compete each other cannot be excluded; whether it was resource or space competition, changes in microbiota or immune mediated changes that benefited co-infected mice in our study remains to be elucidated.

Our expectation was that co-infection would also have a detrimental effect to tolerance against *H. bakeri*. However, co-infected mice tended to be more tolerant compared to mice receiving the *H. bakeri* only infection. In our study, tolerance was estimated in four different ways; the basis of tolerance in hosts is relatively poorly understood, and as such, it is best to consider several different traits of host fitness and pathogen severity in the calculation of tolerance to get a true picture of the nature of host-parasite interaction (Jackson et al. 2014). All four tolerance estimates resulted in similar outcomes, with co-infected mice tended to be more tolerant to *H. bakeri* infection compared to *H. bakeri-*only infected mice. Previous work showed that during a *T. spiralis* and Influenza virus co-infection, mice showed reduced lung pathology and accelerated weight recovery compared to single infected mice (Furze et al. 2006). In that study, viral clearance was not affected by co-infection, which indicates that the positive impact on weight recovery and lung pathology was likely not associated with improved resistance in the co-infected animals. Although tolerance was not calculated as such in that study, the improvement in performance (weight) and fitness (pathology) in the absence of a reduction in pathogen load, are indicative of improved tolerance. The authors speculated that a decrease in inflammation markers observed in the coinfected animals, likely attributed to the ability of *T.spiralis* to suppress inflammation, may be responsible for the reduction in pathology, in the absence of an effect on the resistance (unaffected viral load). In our model, it is possible that the immune response to the TMEV may demonstrate a similar anti-inflammatory ability and improve tolerance in the co-infected mice. Future investigation in these mechanisms would be beneficial to inform decisions on infection control measures.

In conclusion, this study found that the resistance and tolerance response to *H.bakeri* infected C57BL/6 mice can be shifted by exposing the host to a viral pathogen, at the early stages of the helminth infection prior to helminth expulsion. Our study implemented the 3Rs principles in animal research (Lewis 2019), thus using the minimum number of animals necessary. Great care has been taken in the experimental design and the statistical analyses to reduce Type I and Type II errors, e.g. by minimising potential confounding factors not accounted for in the analyses. That said, future experiments with similar experimental designs, possibly involving mice with different genetic backgrounds, are warranted to validate the findings of this study. This proof-of-concept study highlights that co-infection and the order of pathogen infection could influence results of resistance and tolerance investigations and needs to be carefully considered in future studies. The underlying mechanisms of these interactions should be further researched and may be further affected by genetic, physiological, or environmental variation.

## Supplementary Information

Below is the link to the electronic supplementary material.

Supplementary figure 7 (PNG 795 KB)

High Resolution Image (TIF 2.91 MB)

Supplementary File 2 (DOCX 391 KB)

## Data Availability

The datasets used and analysed during the current study are available from the corresponding author on reasonable request.
