# Supplementary figures and images for: Co-infection with a viral pathogen (Theiler’s murine encephalomyelitis virus) tended to improve host tolerance but significantly enhanced resistance to Heligmosomoides bakeri
Source: Vet Res Commun. 2026 May 1;50(4):296. doi: 10.1007/s11259-026-11217-0 (PMC13134990; doi:10.1007/s11259-026-11217-0)

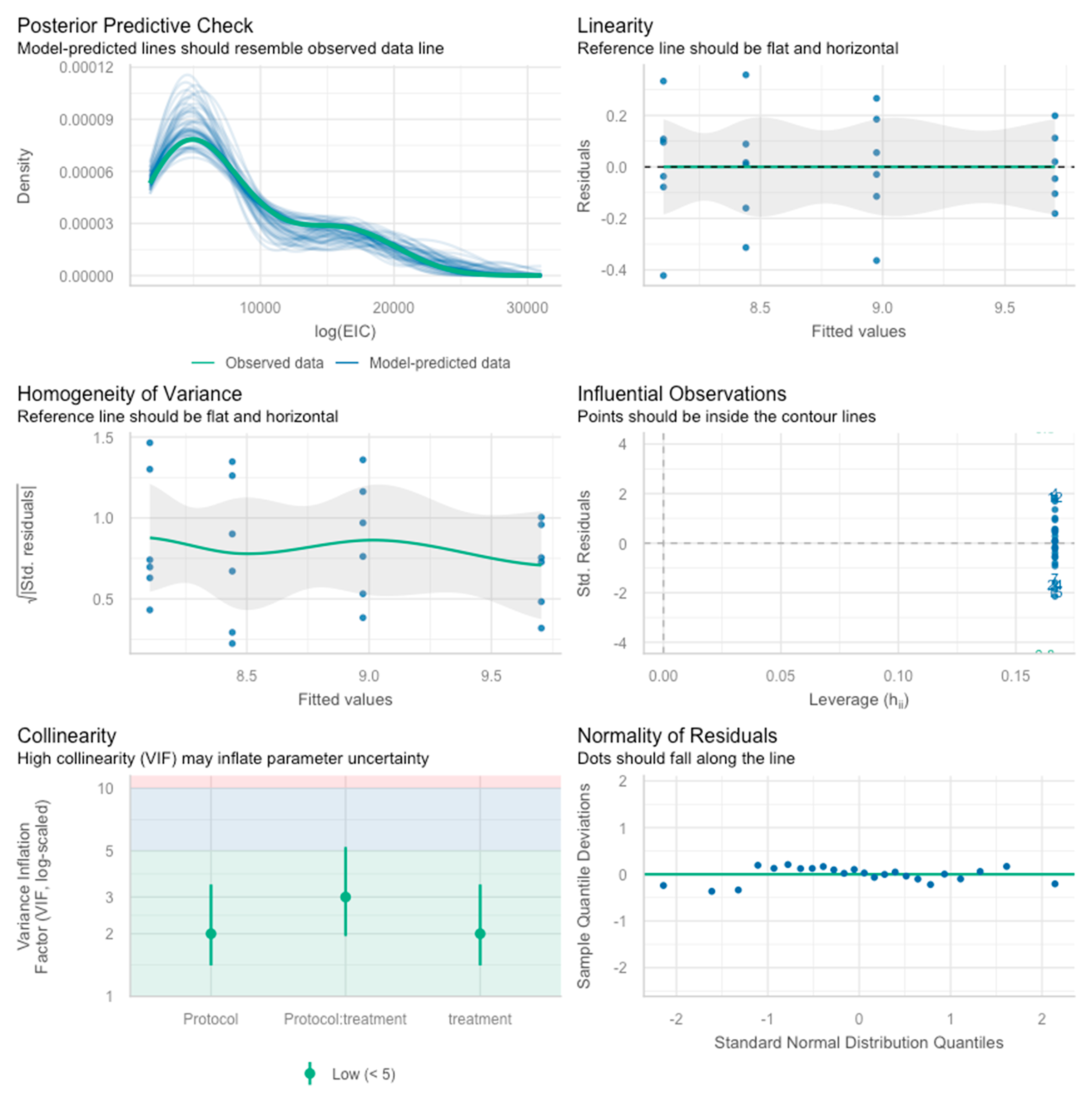

Supplement: Supplementary file 1 — (PNG 795 KB) [file 11259_2026_11217_Fig7_ESM.png]

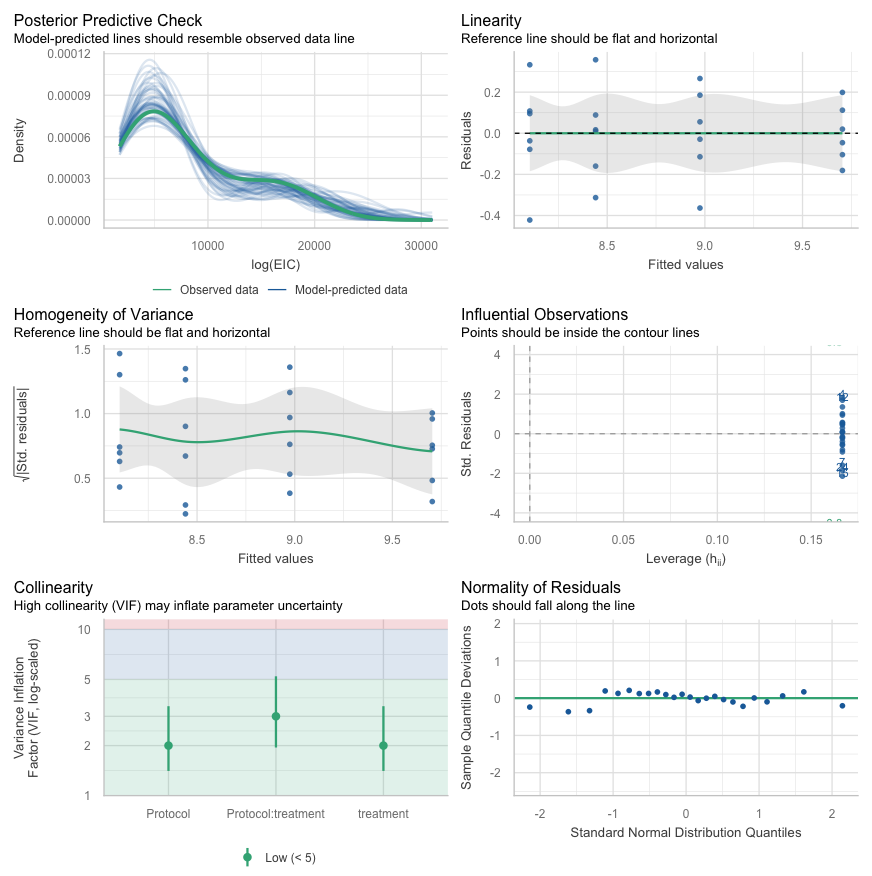

Supplement: Supplementary file 2 — High Resolution Image (TIF 2.91 MB) [file 11259_2026_11217_MOESM1_ESM.tiff]
