# Supplementary material for: Co-infection with a viral pathogen (Theiler’s murine encephalomyelitis virus) tended to improve host tolerance but significantly enhanced resistance to Heligmosomoides bakeri
Source: Vet Res Commun. 2026 May 1;50(4):296. doi: 10.1007/s11259-026-11217-0 (PMC13134990; doi:10.1007/s11259-026-11217-0)
Supplement: Supplementary file 3 — Supplementary File 2 (DOCX 391 KB) [file 11259_2026_11217_MOESM2_ESM.docx]

**Supplementary S2**

**Model fit for EIC**

***Comparison of 2 lmer (Linear Mixed) models:***

Model1: cage as random effect; with interaction

Model2: Cage as a random effect; no interaction


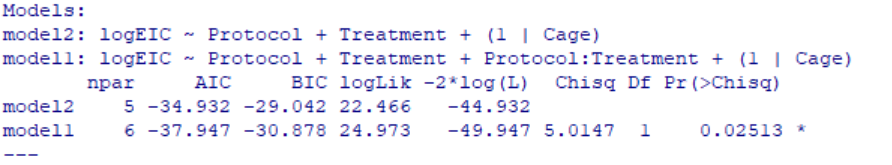
Result: Model 1 is the better model based on AIC, BIC and -2LogL. The interaction is significant. However, model 1 is “singular”. The random effect is estimated as zero. Removal of the interaction term in Model 2 the singular warning disappears.

***Comparison of lm (Linear, no random effect) models:***

Note: There were 2 other models with cage as a fixed effect in the model. However, cage is not estimated if it is included in the model as a fixed effect. Cage is essentially explained by protocol and treatment

Model3: No random effect; with interaction

Model4: No random effect; no interaction


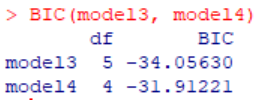

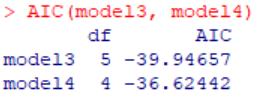
Results: Model 3 is the better model. The interaction is significant.

***Comparison of lmer and lm models:***

Comparison of model 1 (with cage as a random effect) and model 3 (no random effect). The interaction is in both models. This is a comparison of a random effects model and a linear model. To do this the mixed model had to be run as a maximum likelihood (REML=FALSE). It is my understanding this is OK but there may still be some issues with comparison.

Results: The model coefficients are the same. The interaction is significant in both. By both the AIC and the BIC, model 3 (linear, no random effect, with interaction) is best.


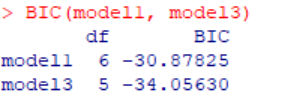

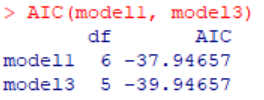


,

Given that the parameters are the same (standard errors are different) the lm model was deemed fit (lowest AIC value) and it has been tested for cage as a random effect and it was zero so not needed in the model.

**Model Fit for Total worm models**

***Comparison of 2 lmer (Linear Mixed) models:***

Model5: Cage as random effect + interaction

Model6: Cage as a random effect; no interaction


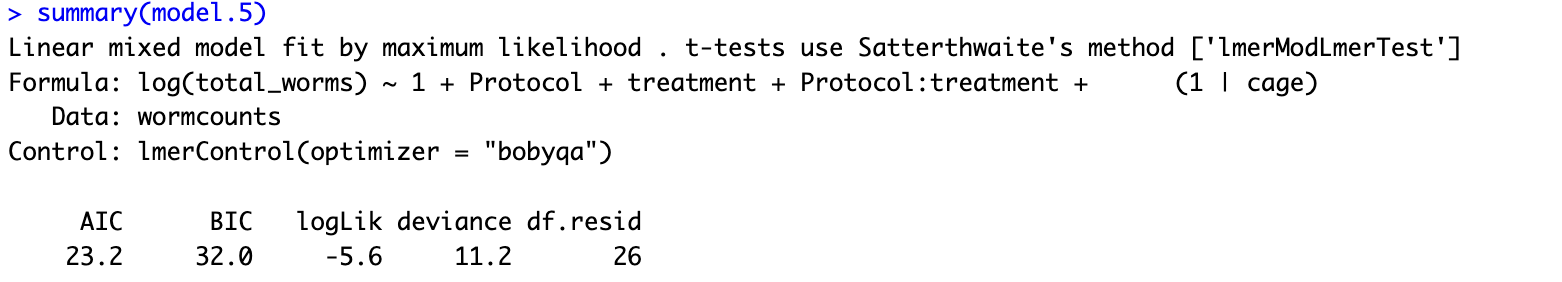


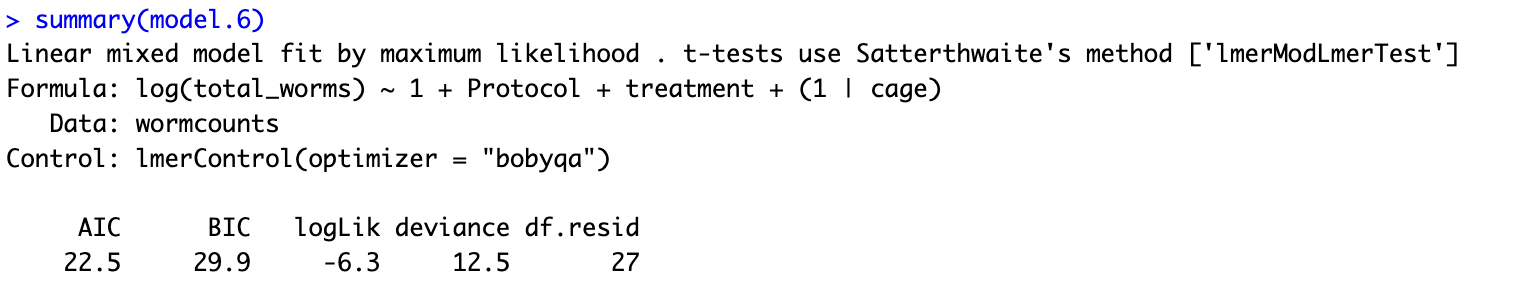


Result: Model 6 is the better model based on AIC (barely) and BIC. The -2LogL was not significant. The interaction is not significant. However, both model 5 and model 6 are “singular”. The random effect is estimated as zero.

***Comparison of lm (Linear, no random effect) models:***

Note: There were 2 other models with cage as a fixed effect in the model. However, cage is not estimated if it is included in the model as a fixed effect. Cage is essentially explained by protocol and treatment

Model7: No random effect + interaction

Model8: No random effect no interaction

Results: Model 8 is the better model based on AIC and BIC. The -2logL is not significant. The interaction is not significant.


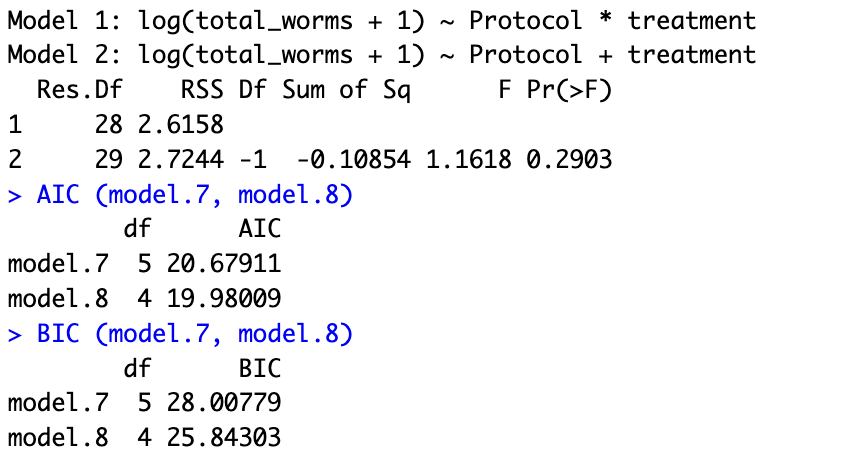


***Comparison of lmer and lm models:***

Comparison of model 6 (with cage as a random effect no interaction) and model 8 (no random effect; no interaction). The interaction is insignificant in both models. This is a comparison of a random effects model and a linear model. To do this the mixed model had to be run as a maximum likelihood (REML=FALSE).

Results: The model coefficients are the same. The interaction is not significant in both. By both the AIC and the BIC, model 8 (linear, no random effect, no interaction) is best.


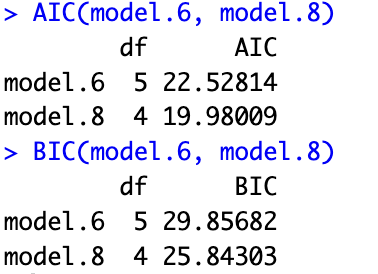


**Table summarising AIC and BIC of different models**

| **Model** | **random** | **Fixed 1** | **Fixed 2** | **Fixed 3** | **AIC** | **BIC** |
| --- | --- | --- | --- | --- | --- | --- |
| **Response variable: logEIC** | | | | | | |
| lmer | Cage | Treatment | Protocol | Treatment*Protocol | -37.947 | -30.878 |
| lmer | Cage | Treatment | Protocol | - | -34.932 | -29.042 |
| **Lm** | **-** | **Treatment** | **Protocol** | **Treatment*Protocol** | **-39.947** | **-34.056** |
| Lm | - | Treatment | Protocol | - | -36.624 | -31.912 |
| **Response variable: totalworms** | | | | | | |
| lmer | Cage | Treatment | Protocol | Treatment*Protocol | 23.22 | 32.01 |
| lmer | Cage | Treatment | Protocol | - | 22.52 | 29.85 |
| Lm | - | Treatment | Protocol | Treatment*Protocol | 20.67 | 28.00 |
| **Lm** | **-** | **Treatment** | **Protocol** | **-** | **19.98** | **25.84** |
| **Response variable: per capita fecundity** | | | | | | |
| lmer | Cage | Treatment | Protocol | Treatment*Protocol | 156.57 | 150.46 |
| lmer | Cage | Treatment | Protocol | - | 154.91 | 149.67 |
| Lm | - | Treatment | Protocol | Treatment*Protocol | 153.18 | 148.66 |
| **Lm** | **-** | **Treatment** | **Protocol** | **-** | **152.66** | **147.24** |

Lmer, R, Linear Mixed model

Lm, R, Linear model

*, interaction

For EIC and total_worms the models with the lowest AIC and BIC is the linear model (no random effect). For EIC the interaction is significant but not for total worms.
